# Sleep Quality Among Older Women and Men in the United States by Sexual Orientation

**DOI:** 10.1093/geroni/igaa057.562

**Published:** 2020-12-16

**Authors:** Adena Galinsky, Karen Fredriksen Goldsen, James Dahlhamer, Tina Norris

**Affiliations:** 1 National Center for Health Statistics, Hyattsville, Maryland, United States; 2 University of Washington, Seattle, Washington, United States

## Abstract

Sleep problems may increase the risk for, and result from, other health problems and negatively impact quality of life. Lesbian, gay, and bisexual older adults report more sleep problems compared to their straight counterparts when such problems are measured in the aggregate (e.g. “one or more of four specific sleep problems”). However, scant national research has examined if specific types of sleep problems vary by sexual orientation among older adults. Using 2015-2018 National Health Interview Survey (NHIS) data, we used logistic regression to separately model five sleep problems among women 50+ and men 50+ (lesbian/gay women: n=377, bisexual women: n=142, straight women: n=33,216; gay men: n=508, bisexual men: n=115, straight men: n=25,998) as functions of sexual orientation, controlling for age, race, education, and income. Sexual minority older adults were more likely than their straight counterparts to have taken sleep medication in the past week (women AOR=2.04, 95% CI:1.55, 2.67; men AOR=1.81, 95% CI:1.36, 2.40). The only other difference by sexual orientation found for men was bisexual older men’s greater likelihood, compared to straight men, of having difficulty falling asleep (AOR=2.02, 95% CI: 1.08, 3.79). Older women did not differ by sexual orientation in difficulty falling asleep, difficulty staying asleep, or waking up not feeling rested for four or more days in the past week, or meeting National Sleep Foundation recommendations for hours of sleep per night, whether lesbian/gay and bisexual women were examined together or disaggregated. Future research may examine why sleep quality only sometimes varies by sexual orientation.

